# Theories and Politics of COVID-19

**DOI:** 10.1177/00027642221132808

**Published:** 2022-11-01

**Authors:** Jeremy Schulz, Noah McClain, Laura Robinson, Juliana Maria Trammel

**Affiliations:** 1 UC Berkeley, Berkeley, CA, USA; 2 Santa Clara University, Santa Clara, CA, USA; 3 Department of Journalism and Mass Communications, Savannah State University, Savannah, GA, USA

**Keywords:** COVID-19, social theory, pandemic politics, solidarity, trauma, social movements

## Abstract

This issue brings theoretically driven analyses to bear on the COVID-19 pandemic, a development which shines a singularly revealing light on some of the most significant political, cultural, and social trends of the 21st century. Leading off with three explicitly theoretical treatments of the pandemic, this issue directs several complementary theoretical lenses at the early stages of the pandemic as it unfolded in the US and Europe. The initial contributions grapple explicitly with the ways in which social conflicts, social solidarities, and social traumas have been refracted—and in many cases magnified—by the pandemic in terms of moral cultures and forms of communication. The focus then shifts to how risk governance during the pandemic operates in different levels and domains of the social architecture as conceptualized in theoretical treatments of social actorhood pioneered by James Coleman. In the second part of the issue, the theme of politic. The upsurge of politically distinctive protests in the United States related to pandemic restrictions—as well as social and racial inequalities rendered visible by the pandemic—is the subject of the first piece. The final article explores the historical specificity of the many popular mobilizations in relation to the pandemic across the globe and across the political spectrum. In this article, we see how the popular mobilizations vary not only in terms of their political orientation, but in their general orientation toward information and authority—increasingly crucial issues in a world facing a trust deficit.

This issue of the ongoing series on the Covid-19 pandemic approaches the crisis from the standpoints of social theory and politics, complementing the other issues in the series. In the first part of this issue we begin with a wide-ranging overview of the pandemic’s effects on the cultural psyche of the US, especially the tight-knit smaller communities often identified with the “heartland” of the country. Building on neo-Durkheimian theories of moral solidarity, theories of collective trauma, risk society theory, and historically informed understandings of disease crises, the article “Disintegration in the Age of COVID-19: Biological Contamination, Social Danger, and the Search for Solidarity” lays out an argument about the many ways in which the pandemic undermines the togetherness so central to social life in the small-town communities making up this region. More than the relatively anonymous densely settled coastal cities which have taken center stage in the pandemic, small-town communities are bound together through face-to-face contacts and in-person interaction within kin and nonkin networks. As such, they are particularly susceptible to the restrictions put in place to combat the spread of COVID-19. Further, due to the long-term erosion of their economic foundations and social fabrics, these communities cannot cope with the economic fallout emerging from the pandemic and the measures taken to contain it. Most significantly, in a cultural context where social life is deeply politicized and moralized, the nebulous character of the pandemic—an agent-less and omnipresent threat without an easily identifiable source—renders it particularly hazardous to moral solidarity.

The following contribution entitled “A Social Diagnosis of Digitally Mediated COVID-19 Trauma” also deals with the trauma stemming from the pandemic from a theoretically driven perspective. However, in this article, the trauma is explored from an experiential perspective, as secondary trauma conveyed through the mediated communication channels of a society continuously engaged in what Castells calls mass self-communication. At the same time, this treatment of secondary trauma—analytically framed as a social condition amenable to social diagnosis—takes into account another form of trauma connected to the exclusion from communication and engagement due to digital exclusion. In a curious paradox, while large majorities of the populations of developed countries experience the trauma of strangers and others in their own circles through media channels of all kinds, others who lack adequate access to digital communication channels endure trauma arising from their inability to engage with individuals and institutions in ways that minimize their exposure to health and economic risk. This situation results in what the authors call a “digital trauma paradox.” In this paradoxical situation, many suffer trauma because of the unrelenting overexposure to others’ hardships mediated through social media platforms and the like. At the same time, a significant part of the population suffers trauma on account of the social isolation and access impediments which prevent them from safely communicating with friends and loved ones or otherwise participating in the social and economic life of society through digital communication channels.

The third article takes up the theme of social solidarity during the pandemic in a country which was one of the first to experience it, namely Italy. In this piece, entitled “Between Online and Offline Solidarity: Lessons Learned from the Coronavirus Outbreak in Italy,” the focus is on the various kinds of mediated solidarity generated across Italy during the pandemic through the use of social media platforms, specifically Twitter. Revisiting the Durkheimian distinction between mechanical and organic solidarity, the study delves into four specific case studies in which Italians joined together virtually in the course of the pandemic. In some of these cases, the effect of the mediated communications was to underscore the commonalities among the various individuals who all shared similar experiences of threat and anxiety, leading to the strengthening of group-level cohesion. In other cases, the communications were geared toward expressing and offering support specifically oriented to the most vulnerable members of society, underlining the social complementarities between the more and less protected members of society, and implicating more organic forms of social solidarity. By elucidating these episodes of virtual solidarity in the midst of the pandemic crisis, this account advances our understanding of the complex forms which high mediated forms of solidarity may assume.

This initial section closes with a theoretical treatment of the pandemic from the perspective of risk governance as opposed to solidarity. Ranging across a variety of social contexts and settings, the study has pinpointed the ways in which governance roles intersect with different forms of social actorhood. This article formulates a general theory of risk governance as a function of the types of social actors involved in distinctive social orders and their corresponding social actors as conceptualized in the pioneering work of James Coleman. In this piece, entitled “Risk Governance in the Early Pandemic: Governance Roles and Coleman’s Taxonomy of Social Actors,” a general theoretical account of risk governance is presented in relation to Coleman’s central distinctions between so-called “natural persons” and individuals acting as proxies for complex corporate social actors such as private employers, customer-facing retailers, and sovereign governmental entities. In tandem with Coleman’s taxonomy of social actors, the article presents a three-fold classification of governance roles (target, implementer, and initiator) which can be occupied by any of these social actors in specific social settings. In this way, the study is able to distinguish specific governance schemes in terms of how they mobilize natural persons and corporate agents as targets, implementers, and initiators of risk governance measures in settings such as private households, employment settings, public-facing retail settings, and institutions of higher education. The case study exposition has shown that Coleman’s theory of social actorhood remains theoretically and conceptually more valuable than ever, given the complications of risk mitigation and governance across the multiple tiers of a highly differentiated society.

In the following section of the issue, the focus turns from theorizing the pandemic to analyzing the politics of the pandemic. This part of the issue starts out with an inquiry into the public protests which have rocked the streets of major and minor U.S. cities during the pivotal year of 2020. The study trains its gaze at what might first appear as a paradoxical feature of the pandemic; namely, that it led state and municipal governments to enact restrictions on public gatherings, and yet triggered waves of public protests at the same time. The authors of this article, entitled “Protest during a Pandemic: How COVID-19 Affected Social Movements in 2020,” address this apparent paradox by arguing that, first, restrictions on movement and gathering in public areas were unevenly enacted and enforced and many people were able to move freely in such spaces. Second, because of the lockdowns and quarantines, coupled with the ubiquitous access to social media and online new platforms, aspiring activists could organize protests easily and rapidly. Finally, the issues which the pandemic brought to the surface were singularly responsible for galvanizing individuals across the political spectrum from the left to the right, depending on the issue. Whereas left-wing protesters came out to protest socioeconomic and racial injustices and inequalities, right-wing protesters took to the streets to contend against government-initiated public health restrictions. In a year of profound crisis which in many ways limited individuals’ experience of the wider world, public protests became an evermore visible part of the nation’s political life.

In the final contribution, the focus turns to a comparative study of social movements during the pandemic across the Global North, including Western Europe, East Asia, and North America. In surveying regions such as the United Kingdom, Hong Kong, Taiwan, and Germany, the study entitled “Mobilizing During the COVID-19 Pandemic: From Democratic Innovation to the Weaponizations of Disinformation,” dissects the highly divergent stances and tactics exhibited by recent social movements with a public presence. Whereas some social movements acted in order to shore up what they perceived as inadequate governmental responses to the pandemic—in Hong Kong for example—in other countries, highly visible social movements surfaced to oppose government-initiated attempts to mitigate or contain the pandemic. Interestingly, such movements emerged mostly in Western Europe and North America. Both pro-mitigation and “COVID negationist” protests made heavy use of online channels for information dissemination and protest organizing. Social movements—particularly on the right—contested official claims about the nature of the pandemic and the mitigation measures needed to contain it, setting in motion a struggle over what counted as truth and who could be counted on to provide reliable information. In this way, the social movements spawned by the pandemic crisis signaled a new chapter in social movements in which disputes over the reliability and authoritativeness of information played important roles alongside more traditional strictly political arenas of contestation.

Taken together, these theoretical and empirical studies break new ground in the study of a pandemic which continues to stretch on into its third year. Surveying the early stages of the pandemic from numerous complementary angles of vision, the articles hone in on some of the most fundamental consequences of this sudden and extraordinarily disruptive worldwide crisis from a variety of theoretical perspectives. While some of the contributions seize on the ways in which the crisis has exploited vulnerabilities in societies and individuals, others grapple with the unexpected consequences of a crisis which has exposed to view the challenges of pervasive social trauma, moral and cultural fragmentation and erosion, political polarization, governance deficits, and the loss of trust of basic social institutions. Thus, the world faces this profound test of its resilience posed by the pandemic with weak social cohesion, widespread trauma, loss of institutional trust, and governance deficits. At the same time, however, the issue does give reason for some optimism, inasmuch as spontaneous upwellings of social solidarity and mutual cooperation have materialized in the face of the dire predicaments which have threatened the life, livelihoods, and liberties of so many during the pandemic.

